# Co-Occurrence of Autism Spectrum Disorder and Achondroplasia

**DOI:** 10.3389/fpsyt.2019.00450

**Published:** 2019-06-26

**Authors:** Angel Belle Cheng Dy, Lourdes Sumpaico Tanchanco

**Affiliations:** ^1^School of Medicine and Public Health, Ateneo de Manila University, Pasig City, Philippines; ^2^MedMom Institute for Human Development, Pasig City, Philippines

**Keywords:** achondroplasia, autism spectrum disorder, neurodevelopmental disorders, developmental delays

## Abstract

Autism spectrum disorder (ASD) and achondroplasia are common disorders on their own. However, this case of co-occurrence in the same patient has not yet been reported in literature except for a hypothesized statistical probability based on prevalence studies stating that two to five in 10 million children could have the probability of having both conditions occurring simultaneously. Achondroplasia typically presents with motor delays and difficulties that are related to musculoskeletal impairments that can affect self-care, mobility, and social cognition; however, the presence of delays in other domains of development, particularly in social communication, raises a suspicion of a co-occurring autism spectrum disorder. The content of this report reviews the common delays and difficulties seen in children with achondroplasia and those with autism spectrum disorder and describes the presence of both in the child presented in this case.

## Background

Autism spectrum disorder (ASD) is a developmental disorder that manifests as difficulties with social communication and interactions and restrictive or repetitive behaviors or interests (1). Achondroplasia is a genetic disorder with characteristic phenotypic presentation of disproportionate short stature, craniofacial and skeletal abnormalities, and motor developmental delays (2). Autism spectrum disorder and achondroplasia are common disorders on their own. However, the co-occurrence in the same patient has not yet been reported in literature except for a hypothesized statistical probability based on prevalence studies stating that two to five in 10 million children could have the probability of having both conditions occurring simultaneously (3).

### Case Presentation

The case is a female, Filipino child born at 37 weeks’ age of gestation. Her skeletal dysplasia was identified on fetal anomaly scanning during pregnancy and was diagnosed with achondroplasia after birth.

She was the third and youngest child to a 36-year-old mother and a 37-year-old father. She has a healthy and neurotypical older sister and brother. The parents were not consanguineous and have no known genetic abnormalities in their family history. Her birth weight was 1,390 g (less than the third percentile, World Health Organization (WHO)), birth length was 40 cm (less than the third percentile, WHO), and head circumference was 32 cm (third to 15th percentile, WHO). Her physical features at birth were described as having proportionately small frontal bossing and dolichocephaly with infraorbital creases. She had a relatively small thoracic cage, which was proportionate to her size. There were no gross rhizomelia or acromelic shortening of limbs. She had bilateral simian creases, bilateral fifth digit midphalanx hypoplasia, and hypoplastic toenails. External genitalia were grossly female.

Peri-natal care included 17-day confinement in the neonatal intensive care unit for hyperbilirubinemia and antibiotic treatment with amikacin and ampicillin for suspected sepsis. Newborn screening and otoacoustic emission tests were normal. She was breastfed for 2 months, after which she was started on formula milk. She has frequent respiratory infections but does not have a history of recurrent ear infections. She also had an exposure to tuberculosis, which was managed with isoniazid for 6 months. Bone aging reports were normal.

At the age of 6 months, delays in motor, language, socio-emotional, and cognitive development were observed. Her fine motor skills included visually tracking, grasping a rattle, and holding her hands together but not yet regarding and reaching for objects. Her gross motor skills were almost of a newborn wherein she was not able to hold up or lift her head. Her language skills included vocalizations, turning to sounds and voices, and laughing but did not yet imitate speech sounds or babbled. Her personal-social skills included regarding faces and smiling responsively and spontaneously but not yet regarding her own hands or feeding herself. Anthropometric measurements were as follows: body weight was 4,100 g (less than the third percentile, WHO), body length was 53 cm (less than the third percentile, WHO), and head circumference was 42.5 cm (50th percentile, WHO). Physical examination revealed universal hypotonia, with a preference for the right hand.

At the age of 17 months, she had improvements in her developmental skills with more motor imitations, vocalizations, and babbling and the ability to follow simple commands. Physical examination continued to show poor muscle tone, flat foot, and joint laxity. She had difficulty feeding as she choked on solid food and had frequent regurgitation. A gastroenterologist assessed her to have gastroesophageal reflux due to hypotonia. She was advised to have slower, smaller feeding to help in her digestion. The issues with reflux resolved by 30 months of age.

In the next 2 years, the child continued receiving physical, occupational, and speech therapy. **Table 2** presents her developmental skills acquisition. It is compared with the skills acquisition of other children with achondroplasia in a study by Ireland et al.

By the age of 27 months, her parents began noticing her poor social skills such as fleeting eye contact, not turning to her name being called, and not pointing or requesting for her needs. Despite undergoing early intervention services, she continued to demonstrate delays in all developmental domains. A summary of her progress and developmental quotient using the Griffith Mental Development Scales is presented in **Table 1**.

**Table 1:** Patient’s performance on the Griffith Mental Development Scales.

	18 months of age	Developmental quotient	39 months of age	Developmental quotient
Locomotor	12 months	67	19 months	49
Personal–social	10 months	56	12 months	30
Hearing and speech	8 months	44	10 months	27
Eye–hand coordination	10 months	55	11 months	28
Performance	11 months	61	15 months	38

At 39 months, she meets the DSM-5 criteria for autism spectrum disorder, manifested by her delays in communication and social skills. She also presented with repetitive and stereotypic behaviors such as a fascination of shadows and hand flapping and would demonstrate repetitive tapping of objects (i.e., pencil) and her hands against hard surfaces like the table.

The Autism Diagnostic Observation Schedule (ADOS-2), Module 1, was administered at 39 months, and she received an additional diagnosis of autism spectrum disorder. The Autism Diagnostic Observation Schedule is a semi-structured standardized assessment of communication, reciprocal social interaction, play, and imaginative use of materials. Module 1 of the assessment was used, which consisted of 10 activities that focused on playful use of toys and other concrete materials. The child’s communication score was 6 (cut-off = 4), and social interaction was 8 (cut-off = 7), which both reached the cut-off for autism.

Management of her developmental delays and behavioral concerns continues to be targeted through speech and language therapies that focus on pre-language skills, pragmatics, and communication of her needs and wants. She also continues occupational therapy and physical therapy to address her motor skills, activities of daily living, and sensory processing concerns. She receives special education with focus on self-regulation and engagement, and behavior modifications to allow her to be involved in learning activities.

## Discussion

### Predispositions and Risk Factors

Although genetic studies were not performed in this patient, achondroplasia may be a cryptogenic cause for her presentation of autism. Approximately 40% of children with autism are diagnosed with other co-morbidities such as epilepsy, neuropsychiatric disorders, attention deficit/hyperactivity disorder, and Tourette syndrome. A term “secondary” ASD has been coined to refer to cases of identifiable syndromes or medical disorders known to be associated with ASD, and these are seen in approximately 6% of confirmed cases. Aside from these, dysmorphic features are seen in some cases of ASD that may indicate genetic syndromes as well (4, 5).

There are a number of genes that are associated with ASD presentation. A study by M.R. Herbert et al. (2006) listed 135 environmentally susceptible genes that showed an overlap with autism linkage regions that may not have been studied yet in relation to autism itself. One of these included the FGFR3 gene for achondroplasia (6). Even though it is merely part of the extensive listing of genes, it highlights that multiple genetic and environmental mechanisms could be playing a role in the presentation of autism in this case.

The parents of this child are both in their mid-30s and may be additional risk factors for her condition. Increased paternal age has been associated as a contributing factor to some diseases and disorders, including both achondroplasia and ASD (7, 8). Increased maternal age over 35 years has also been associated as a risk for having a child with ASD (9).

Abnormal presentation, fetal distress, small for gestational age, low birth weight, feeding difficulties, and hyperbilirubinemia are among the associated risk factors for ASD (10). These risk factors were present in the case.

### Developmental Trajectories

A cohort study by Ireland et al. (11, 12) developed a projection of milestones for children with achondroplasia. Children with achondroplasia commonly have developmental delays in gross motor skills such as lifting their head, rolling over, crawling, and walking. These children develop other pre-ambulation strategies to compensate for their limb shortening and hypotonia such as forward and reverse snowplow or commando crawling as demonstrated by the child in the case (11, 12). In the comparison table with the children diagnosed with achondroplasia in the Ireland et al. (11, 12) study, her motor performance is at par with that of the cohort of children from the study (**Table 2**).

**Table 2:** Age of achievement (months) for gross motor, fine motor, communication, and feeding skills (11, 12).

Milestone	Patient’s age of achievement	Achondroplasia 90th percentile Ireland et al.	Typical development 90th percentile Denver II Test
Gross motor			
Lift head when lying on stomach	9	7	3.5
Roll over	9	9.9	5.2
Reverse snow plough	12	13.8	
Traditional crawling	16	18.3	
Into sitting from lying	15	18.5	10
Into sitting from standing	18	22.8	
Into standing from sitting	20	20	10
Stand holding on	16	20	12
Stand unsupported	18	24	14
Walk holding on	18	22	12.7
Walk independently	20	26	15
Fine motor			
Reach for object	6	6.5	5.5
Pass objects	12	10	7.5
Bang objects together	18	11.1	11
Scribble with crayon	26	20	17
Draw circle	—	36	45
Build tower two blocks	24	20.9	20
Build tower eight blocks	—	32.9	42
Communication			
Smile	6	2.8	2
Babble	18	11.3	9
Wave	18	14.3	14
Say “mama”	—	14	13
Shake head	—	20	
Peek-a-boo	24	13	9.7
1-step request	36	23.5	
Short sentences	—	38.4	
Feeding			
Cup drinking	36	15.6	17
Puree/smooth solids	12	6.5	
Mashed solids	18	10.2	
Finger feeding	36	12.3	7
Self-feed with spoon	—	22	20

This case presents with delays in fine motor and social skills with slow progress in these developmental domains despite interventions initiated at an early age. The development of fine motor and social skills in children with achondroplasia is expected to be similar with typical development, achieving the 90th percentile for age similar to that of typically developing children (11, 12). The delays in fine motor skills and social skills in our case are better explained by the presence of autism (13).

Early communication skills such as babbling and saying “mama” are likewise expected within typically developing age ranges for children with achondroplasia. However, they take a longer time to achieve the use of single words and two-word combinations but eventually learn them (11, 12). At 39 months of age, our case is expected to use simple sentences, but she is still unable to say or imitate words. She still uses jargons and is only able to follow one-step requests. Therefore, the language delays seen in this patient are uncommon in achondroplasia by her current age; but they can be attributed to the impairments in ASD.

Based on the Ireland et al., (14) study, skills for independence emerge by the fourth and fifth years of life. By 7 years old, children can be independent or have modified independence for self-care skills such as eating and toileting. They are also completely mobile, either independently or with modifications. Social cognition also improves, showing difficulty in comprehension only (11, 12, 14). The musculoskeletal impairments of achondroplasia seen in this patient increase the need for a caregiver’s assistance. It is possible to predict that she will continue to have difficulties in independence for a number of years. However, these may be overcome through continuous intervention in her therapy sessions as well as creation of modifications that allow for independence.

Barriers in the medical and social life course of individuals with achondroplasia include unequal opportunities in education and employment and increase in social isolation, which then influence their financial situation and quality of life (15). It is important to recognize that other difficulties of individuals with achondroplasia would be social in nature, particularly public perceptions that lead to teasing and discrimination. Individuals may have lower self-esteem, less education, lower annual income, and less likely to marry. In their transitions through different life stages, interventions should also include psychotherapy in fostering a positive self-concept to increase quality of life (16).

Anticipatory guidance and regular health supervision will assist in earlier identification of known complications in achondroplasia and includes confirming the diagnosis through radiographic studies, documentation of measurements, medical evaluations, and determining social adjustment (17). This patient benefited from early detection and appropriate monitoring for her disorder through access to a pediatrician and geneticist. She has also been followed up by a developmental-behavioral pediatrician in order to monitor her development, leading to early detection of her developmental delays, which are being addressed through a number of interventions, including speech therapy, occupational therapy, and physical therapy. Multidisciplinary approaches in caring for these types of special cases are necessary to optimize management and care (11, 12).

## Conclusion

Achondroplasia is a genetic disorder with characteristic phenotypic presentation of disproportionate short stature, craniofacial and skeletal abnormalities, and motor developmental delays. Its co-occurrence with autism spectrum disorder has not been reported before. This patient presents with physical features of achondroplasia with the concomitant delays in social communication and stereotypic behaviors seen in autism spectrum disorder. Early intervention is critical in addressing the unique needs of this patient. Collaboration among the intervention team is necessary to optimize her development.

This case report has its limitations. It is unable to generalize to other populations, and the association does not imply there is a cause–effect relationship between autism and achondroplasia. It could be that the observation is merely a coincidence. Achondroplasia in this case was based on clinical features of the child. More intensive work-up including radiographic studies, documentation of measurements, extensive medical evaluations, and determination of social adjustment, when available, should be used to establish baseline findings. It is recommended that her developmental trajectories be monitored as she grows up.

## Ethics Statement

A written informed consent for writing and publishing this report was obtained from the parent and legal guardian of the individual included.

## Author Contributions

LT performed the clinical assessment and follow-up of the case. AD reviewed related literature. LT and AD reviewed the subject’s medical history and physical presentation. AD drafted the manuscript. LT provided critical revisions to the manuscript and provided the final approval for the document to be submitted for publication.

## Funding

Support for publishing this article is provided by the Ateneo Center for Research and Innovation, Ateneo School of Medicine and Public Health.

## Conflict of Interest Statement

The authors declare that the research was conducted in the absence of any commercial or financial relationships that could be construed as a potential conflict of interest.
